# An Atypical Growth and Maturation Stage of Mandibular Ossifying Fibroma Occurrence Resembling a Different Fibro-Osseous Lesion—Correlation Between Radiological and Histopathological Data

**DOI:** 10.3390/diagnostics15111367

**Published:** 2025-05-29

**Authors:** Kamil Nelke, Klaudiusz Łuczak, Marcelina Plichta, Maciej Janeczek, Agata Małyszek, Piotr Kuropka, Maciej Dobrzyński

**Affiliations:** 1 Maxillo-Facial Surgery Ward, EMC Hospital, Pilczycka 144, 54-144 Wrocław, Poland; 2 Health Department, Angelus Silesius Academy of Applied Sciences in Wałbrzych, Zamkowa 4, 58-300 Wałbrzych, Poland; 3 Faculty of Chemistry, University of Wroclaw, Fryderyka Joliot-Curie 14, 50-300 Wrocław, Poland; 4 Department of Biostructure and Animal Physiology, Wrocław University of Environmental and Life Sciences, Kożuchowska 1, 51-631 Wrocław, Poland; 5 Division of Histology and Embryology, Department of Biostructure and Animal Physiology, Wrocław University of Environmental and Life Sciences, Cypriana K. Norwida 25, 50-375 Wrocław, Poland; 6 Department of Pediatric Dentistry and Preclinical Dentistry, Wrocław Medical University, Krakowska 26, 50-425 Wrocław, Poland; maciej.dobrzynski@umw.edu.pl

**Keywords:** ossifying fibroma, mandible, jaw tumor, allogenic bone, bone lesion

## Abstract

The occurrence of osseous, fibrous, and fibro-osseous lesions in the jaw bones might pose challenges for accurate diagnosis and the selection of the best therapeutic approach. Certain radiolucent, radiopaque, or mixed-origin lesions can look very similar to other bone lesions, because of the stages of their growth, calcification, maturation, and possible local factors affecting the lesion. Ossifying fibroma (OsF, OF) is a type of fibro-osseous lesion, whose radiological characteristics might sometimes be uncertain. It may appear on classic radiographs and cone beam computed tomography as a radiolucent/radiopaque lesion with calcification bodies or a shape with a cloud-like appearance. The appearance is mostly related to the lesion’s maturation level, calcification stage, and number of fibrous elements. Diagnosis might be challenging. Its histopathological evaluation reveals a combination of mineralized and fibrous connective tissues in the mass. From a radiological point of view, because of the tumor’s various stages of bone remodeling, formation, and resorption, diagnosis might be troublesome. Different diagnoses should include cemento-osseous dysplasia, fibrous dysplasia, or cementoblastoma. A biopsy could provide an accurate histopathological examination, improving diagnosis and influencing later surgical approaches. Regardless of the final specimen evaluation, surgery is the treatment of choice. The authors would like to present the correlation between radiological and histopathological data in tumor treatment outcomes.

**Figure 1:**
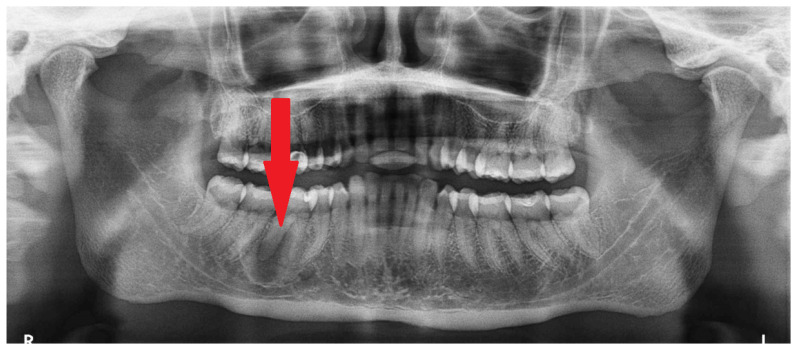
The occurrence of different irregular radiolucent areas on panoramic radiographs (panx) is quite a typical finding. Most are related to periapical teeth lesions, granulomas, odontogenic cysts, or other conditions. The occurrence of osseous, fibrous, or fibro-osseous lesions (cemento-osseous dysplasia, ossifying fibromas (their types psammomatoid, juvenile), and fibrous dysplasia (DF) in the mandible requires improved diagnostics with CBCT—cone beam computed tomography. A classic panoramic radiograph has many limitations in fully investigating each bone lesion in the jaw. Assessing the status of dental roots (displaced, resorption), the cortical buccal and lingual plates (expansion, swelling, erosion), the presence of periosteal reaction or elevation, and the shape, size, and status of adjacent teeth might be quite limited. The occurrence of ossifying fibroma (OSF) might be misdiagnosed as certain bone dysplasias, odontogenic cysts, or cemento-osseous dysplasia (COD) at various stages of maturation and calcification. It may also be mistaken for static bone cavities (SBCs) or even overlooked on panx. In some cases, similar bone cavities might be associated with other bone pathologies like brown tumors in hyperparathyroidism or other thyroid, parathyroid, and pituitary gland anomalies related to calcium–phosphorus metabolism [1,2,3]. The first step should always include improved diagnostics, followed by a scheduled biopsy—such as incisional biopsy—or lesion observation. Improved diagnostics may include SPECT (single-photon emission computed tomography) or, to exclude any metabolic disorders, a complete blood panel that examines calcium–phosphorus metabolites (serum calcium concentration (Ca), parathyroid hormone (PTH), active vitamin D (1,25(OH)2D), magnesium, blood phosphorus levels, bone alkaline phosphatase, thyroid hormones such as thyroxine (T4) and triiodothyronine (T3), and TSH [1,2,3]). The presented panx shows a radiolucent, irregular area under tooth 46 (the lower right first molar) without a clear border (red arrow), adjacent to the superior part of the mandibular canal, without any clear teeth resorptions or displacements.

**Figure 2:**
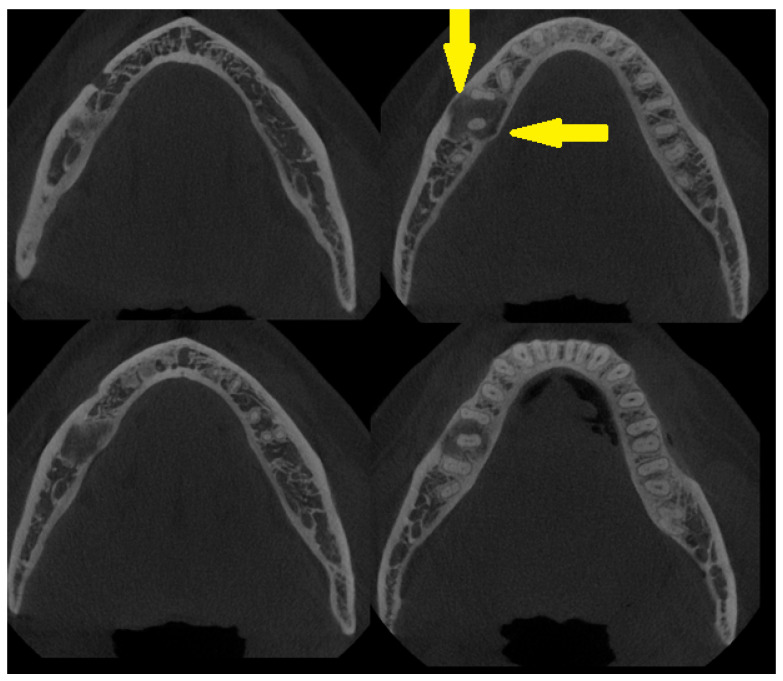
An axial CBCT view of the lesion—cortical swelling is barely noticeable; however, thinning of the buccal cortical plate can be seen along with totally intact roots of the first molar and second right premolar teeth (yellow arrows). The course of the mandibular canal was not disturbed, and clinically, no neurological symptoms from the inferior alveolar nerve were present (IAN). Because the lesion surrounded the first right molar and had some atypical bone borders (without tooth mobility, pain, or abnormal response to cold stimulus), and the lesion itself had a cloudy appearance without any clear signs of calcifications characteristic of fibrous dysplasia (DF) or any of the three growth/maturation stages characteristic of a COD lesion (cemento-osseous dysplasia), a decision was made to conduct a biopsy [4,5]. From a surgical point of view, COD small lesions might be observed clinically or radiologically or, in rare cases, scheduled for an incisional biopsy to remove and evaluate them. Some serious clinical implications start to arise when the lesion becomes larger and exhibits a non-characteristic radiographic appearance on CBCT. Due to the absence of general and local symptoms and the accidental discovery of the bone lesion, after the CBCT, a biopsy was scheduled, and the diagnosis of OSF was confirmed. On the other hand, because of the similarities among COD, DF, and OSF, in classic, non-growing, and self-limiting bone lesions, observation combined with tooth vitality testing is sufficient for the local control of the disease. Radiological signs like root resorption, cortical spread, bone asymmetry, and others might require some type of surgical intervention.

**Figure 3:**
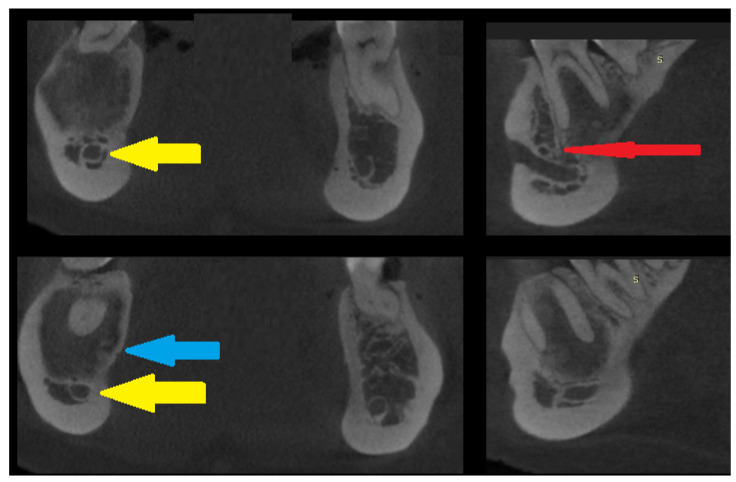
CBCT scans in the coronal and sagittal views—slight bone swelling with cortical thinning (blue arrow) and a non-displaced mandibular canal (yellow arrow). A clear root structure without any erosion, resorptions, or other pathology is visible. The red arrow on the sagittal scan reveals a good condition of the nerve and blood vessels entering the periapical area of the first right molar tooth in the mandible. This cloud-like appearance is characteristic of many lesions in radiologic studies [1,2,3,4,5]. No extracortical bone spread is visible. Identifying the extent of lesion calcification or the occurrences of bone- or tooth-like structures within the cavity could improve diagnostic accuracy. A study conducted by Gennaro et al. confirmed that diagnosis is challenging, but the main goal should always be predicting the right outcome for the patient. Each lesion in the jaw bones requires careful evaluation of the cortical bone, potential calcifications, presence of tooth-like structures, the status of the periosteum, and the formation of any new bones. On the other hand, a study by Merva Soluk-Tekkesin et al. indicated that the most common radiological feature found in their study was a mixed radiolucent/radiopaque internal structure [4]. The cloud-like radiological appearance made it very hard to distinguish between types of bone and fibro-osseous lesions and to determine the most suitable approach. This case underlines how CBCT and biopsy might, in some cases, influence the selection of a proper surgical approach. Many radiological scans of known bone lesions exhibit typical characteristics for each lesion. In this case, radiological differentiation of the lesion was very challenging, especially on PANX and CBCT. This OSF has two different radiological appearances and unclear borders. Quite often, only thorough histopathological evaluation can accurately reveal the different patterns of bone growth, maturation, and calcification that confirm the diagnosis of OsF. Abbreviation—S: surgery side (right).

**Figure 4:**
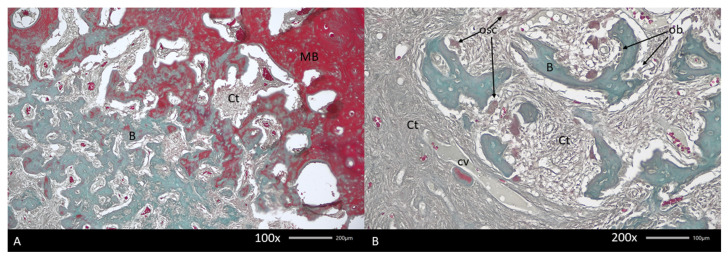
Histopathological image of biopsy taken from a mandibular fibro-osseous tumor. Fibrous tissue in woven bone islet spaces and different forms of bone formation and resorption are noticeable. Various forms of bone maturation and reorganization are common. In the presented figures, the gradual change in bone structure is from mineralized (MB) to poorly mineralized bone (**A**). In both areas, connective tissue (Ct) at different levels of density is visible. Note the differences in bone structure between mineralized and unmineralized bone. (**B**) Typical image of soft tissues at the border between different radiological densities, containing islets of newly formed woven bone. Between collagen fibers, only capillaries are noted (cv). Note the presence of osteoblasts (obs) and multinuclear osteoclasts (oscs) attached to the same bone fragment. Mallory stain.

**Figure 5:**
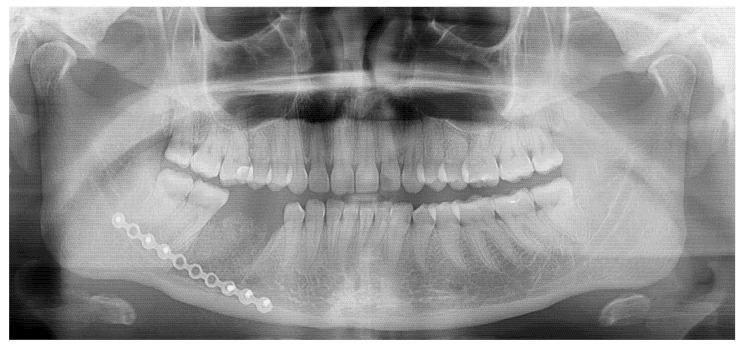
A control postoperative panx—a very good surgical result. The final surgical step consisted of en bloc resection of the mandibular segment containing two teeth (46,45) while maintaining a thin portion of the lower mandibular basis. The inferior alveolar nerve was exposed but left intact, lying at the bottom of the right mandibular basis, surrounded by a three-wall bone defect. Surgical burrs were used additionally to perform an ostectomy of all bone margins. The final defect was packed with an allogenic bone graft and the patient’s blood–IPRF mixture. A titanium 2.0 plate (Medartis, Basel, Switzerland) and six titanium 2.0 7/8 mm long (Medartis, Basel, Switzerland) screws were used to reduce the possibility of iatrogenic mandibular fracture. The postoperative period was uneventful. A layer-by-layer suturing was used to fully cover the bone defect. Disturbances in the function of the inferior alveolar nerve were noted. Oral medication consisted of Neurovit 0.32 mg two times per day for 50 days (1 tablet containing 100 mg vitamin B1, 200 mg vit. B6, and 0,2 mg vit. B12; 100 tablets; G.L. Pharma, Gerot-Lannah, Wien, Austria) and Thionerv-600 once a day for 30 days (600 mg alpha-lipoic acid; Solinea, Elizówka, Poland). This regimen improved nerve function in four months. A postoperative radiograph additionally visualizes the removed bone block with OSF and two adjacent teeth embedded into the lesion. The final clinical remark points out that radiological studies have some limitations in fully establishing the type of fibro-osseous lesion in the jaw bones; however, when combining histopathology with CT/CBCT imaging, the result is easy to predict, without any misdiagnosis. Other therapeutic options might include lesion observation; however, there is a risk of lesion enlargement and potential pathological mandibular fracture.

**Figure 6:**
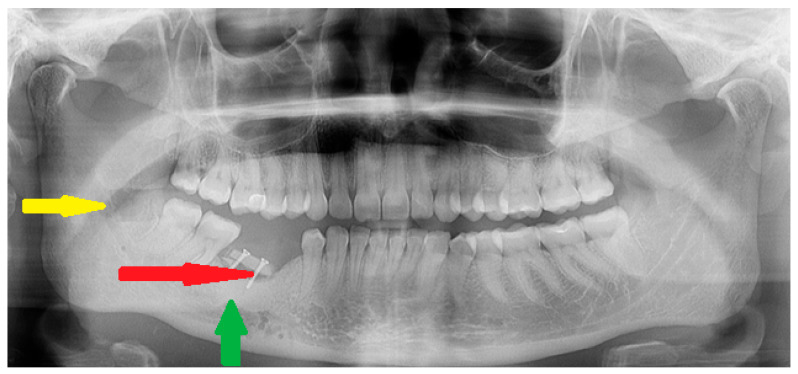
Final results at 24 months after surgery. Due to superior resorption of the previously placed allogenic bone, a decision was made to simultaneously remove the titanium plate, take a blind biopsy, and add some autologous bone graft transfer (red arrow) from the right mandible ramus (yellow arrow). This method was used to improve the height and diameter of the defect for future dental implant placement. Titanium bone grafting screws that were 1.2 mm/11 mm long were used to stabilize the grafted bone (Titamed, bone grafting screws PHI system, Kontich, Belgium). Significant bone remodeling after allogenic bone graft usage is evident (green arrow). Currently, the patient is free of the disease and awaits dental implant therapy.

## Data Availability

The datasets used and/or analyzed during the current study are available from the corresponding author upon reasonable request.

## References

[1] Sugino N., Kuroiwa H., Shimada K., Sato T., Taguchi A. (2024). A Case of an Ossifying Fibroma of the Mandible Suspected as a Static Bone Cavity. Cureus.

[2] Collins L.H.C., Zegalie N.F.T., Sassoon I., Speight P.M. (2023). A Clinical, Radiological and Histopathological Review of 74 Ossifying Fibromas. Head Neck Pathol..

[3] Seifert M., Nelke K.H., Noczyńska A., Łysenko L., Kubacka M., Gerber H. (2015). Bone markers in craniofacial bone deformations and dysplasias. Postepy Hig. Med. Dosw. (Online).

[4] Gennaro P., Gennari L., Latini L., Cavati G., Vannucchi M., Giovannetti F., Cascino F. (2024). Maxillofacial Bone Involvement in Fibro-Osseous Lesions: Emphasizing the Significance of Differential Diagnosis. J. Clin. Med..

[5] Soluk-Tekkesin M., Sinanoglu A., Selvi F., Cakir Karabas H., Aksakalli N. (2022). The importance of clinical and radiological findings for the definitive histopathologic diagnosis of benign fibro-osseous lesions of the jaws: Study of 276 cases. J. Stomatol. Oral Maxillofac. Surg..

